# Comparison of In Vitro Efficacy of Six Disinfectants on the Hatching of Larval Eggs of *Toxocara canis*

**DOI:** 10.18502/ijpa.v15i3.4195

**Published:** 2020

**Authors:** Camilo ROMERO, Rafael HEREDIA, Manuel BOLIO, Laura MIRANDA, Laura REYES, Mauricio ARREDONDO, Ariadna FLORES

**Affiliations:** 1. Research Department, DERMAVET Veterinary Hospital, Mexico City, Mexico; 2. Agricultural Sciences and Natural Resources, UAEM Amecameca University Center, Autonomous University of the State of Mexico, State of Mexico, Mexico; 3. Faculty of Veterinary Medicine and Zootechnics, Autonomous University of Yucatan, Mérida, Yucatán, México; 4. Department of Life Sciences, Guanajuato University, Irapuato-Salamanca Campus, Irapuato, Guanajuato, Mexico

**Keywords:** *Toxocara*, Disinfectants effect, Eggs, In vitro

## Abstract

**Background:**

The environmental contamination with *Toxocara canis* eggs increases the risk of dissemination and transmission of the parasite in dogs and paratenic hosts such as humans. We aimed to evaluate different disinfectants to compare their effect on *T. canis* eggs.

**Methods:**

For its realization, 850 embryonated eggs were obtained, which were suspended in a solution of 5% formaldehyde and distilled water in Eppendorf tubes. In the tubes containing the 850 embryonated eggs, researchers was added 0.5 mL of each solution (enzymatic solution, sodium hypochlorite, iodopovidone, quaternary of ammonium, benzalkonium chloride, and super oxidation solution). After mixing, an aliquot was taken, observed under the microscope, and the number of broken eggs counted at different times to find the most effective ovicidal moment.

**Results:**

The enzymatic disinfectant present a significant difference (*P* = 0.05) with 276.06 broken eggs followed by ammonium with 105.20 broken eggs. After 10 min, the ammonium solution was the one that showed a significant difference of 50.50 hatched eggs, followed by the enzymatic 26.80 and hypochlorite 25.00 treatments. After 20 min, the enzymatic solution treatment showed a significant difference with the other solutions showing an increase of 98.80 broken eggs. In the 30 and 40-min times, only the enzymatic treatment showed a significant difference of 334.10 and 381.70 of broken eggs respectively.

**Conclusion:**

The enzymatic solution has the greatest ovicidal effect against the eggs of *T. canis* to present a greater number of broken eggs in a given time between 20 and 40 minutes.

## Introduction

Toxocariasis one of the zoonoses of high medical importance caused by *Toxocara* spp., mainly by the *T. canis* and *T. cati* (1). *T. canis* is an intestinal nematode of worldwide interest for public health, since parasitized dogs and environmental contamination with eggs of this parasite are important risk factors for the spread of the disease in animals and humans (2).

According to the National Institute of Statistics and Geography (INEGI) in Mexico, 23 million dogs and cats are infected with *Toxocara* species (3). Most will excrete eggs of *T. canis* (dogs) and *T. cati* (cats), which can contaminate the environment. This parasite is common in young dogs, which is relevant because puppies contaminated the environment with large numbers of eggs that can embryonated and become infectious to humans and other paratenic hosts in less than a month (4).

This has a high impact on public health, mainly in the tropics and subtropical countries, where treatment for pets and control of the population are limited. In these populations, there will be a higher risk of infection because exposure to the feces of dogs and cats is recognized as one of the factors for the development of zoonotic disease. Most infections by *T. canis* in humans are asymptomatic, but there is possibility of migration of *T. canis* larvae to the eye and central nervous system. Man can acquire *T. canis* infections due to the accidental ingestion of infective larval eggs, most commonly in contaminated soils, water, products, or on contaminated surfaces (5). On the other hand, this parasite has been suggested as an environmental risk component for asthma in children, as well as a factor for idiopathic seizures and intestinal and cutaneous disorders (6, 7).

*T. canis* eggs are highly resistant to harsh environmental conditions and chemical disinfectants that routinely used (4). For its survival and development, *Toxocara* eggs are affected by different causes such as temperature and humidity. Under laboratory conditions, some eggs may reach to the infectious stage in 5–9 days at optimal temperatures. Whereas in the most natural environments, it usually takes 3– 6 weeks for *Toxocara* eggs to become infectious, in cooler temperatures delay development and larvae die when soil temperatures are below 10 ºC (8).

The resistance of *Toxocara* eggs has been tested in studies controlled such as in soil and hair, determining their ability to develop in the hair of dogs, observing that the temperature and moisture factors is responsible for larvae development in eggs, allowing its viability (9). The disinfection of surfaces contaminated with eggs of *T. canis* is important because of the public health problem for humans and pets. Despite this, the information on the de-contamination of *T. canis* eggs in the safety data sheets of pathogens is unclear (10).

Therefore, the objective of this research was to evaluate the effect of different disinfectants like iodopovidone, sodium hypochlorite, enzymatic solution, ammonium chloride, benzalkonium chloride, and super oxidation solution—on eggs of *T. canis in vitro*, as a measure of disinfection and prevention of toxocariasis.

## Methods

### In vitro larvae of Toxocara canis eggs

The adult parasites were kept in distilled water until they were used in the Laboratory of the Animal Veterinary Clinic (CLIVAC) of the Autonomous University of the State of Mexico (UAEM) Amecameca Unit in November 2018. Subsequently, gravid females were selected and placed in a Petri dish with saline. Their uteri were extracted and from this the eggs of *T. canis,* which were suspended in a solution of 5% formaldehyde in Eppendorf tubes to be later centrifuged at 45 gravitational forces (G) for 3 minutes. The tubes were kept at exposure to solar radiation and at room temperature (20 °C). Later they were observed under a microscope at a 40x objective, counting and checking the eggs every 7 days until the larvae were obtained (11). The number of eggs with mobile larvae was counted in a McMaster chamber (12). The egg suspension was adjusted to a concentration of 850 embryonated eggs with mobile larvae (13).

### Disinfectant solutions used

To the tubes containing the 850 embryonated eggs, 0.5 mL of each of the following solutions was added: enzymatic solution (Aniosyme DLT, Laboratoires ANIOS, France), sodium hypochlorite (Cloralex, Alen Industries, Mexico) at 6%, iodopovidone (Poliyodine, Equipos Medicos Quirurgicos S.A. de C.V., Mexico) 8%, quaternary ammonium (QUAT, Alinat, S.R.L. Argentina) 12%, benzalkonium chloride (Antibenzil, GNJ Manufacturing, S.A. de C.V., Mexico) 10% and super oxidation solution with neutral pH (Dectocide SG9, Betelgeux, S.L. España) at 0.002%, as well as a control group with distilled water. After the mixture, an aliquot of 10 microliters was taken, observed under a microscope, and the number of hatched eggs was counted at 10, 20, 30, 40, and 1440 minutes to estimate the residual effect of long-term solutions.

### Statistical analysis

The experimental design was completely randomized with six treatments and a control with three replications. The number of hatched eggs *Toxocara* eggs and their respective percentages were analyzed by SAS GLM (14), and the means of the treatments were compared by means of the Tukey range (*P* ≤ 0.05) (1).

## Results

The effects of the disinfectants were determined according to the number of hatched eggs (Table 1), where it was observed that the most effective was the enzymatic one (presenting a significant difference (P = 0.05) of 276.06 hatched eggs in relation to the other treatments), followed by ammonium (105.20), the other treatments did not show significant differences.

**Table 1:** Number and percentage of hatched eggs and effects according of different disinfectants

***Treatments used***									
	***Enzymatic***	***Ammonium***	***Hypochlorite***	***Benzalkonium***	***Iodine***	***Oxidation***	***Water***	***EE***	***CV%***
No. of eggs	276.06^a^	105.20^b^	47.84^c^	29.94^c^	27.22^c^	27.22^c^	3.32^c^	7.41	191.5
Hatched eggs %	32.47^a^	12.37^b^	5.62^c^	3.52^c^	3.20^c^	2.05^c^	0.39^c^	0.87	191.5

abc Mean with different literals present significant difference; EE = standard error; CV = coefficient of variation

The effect of the disinfectants was measured at certain periods of time, in order to determine when each of them would be most effective.

After 10 minutes, ammonium was the one that showed a significant difference of 50.50 broken eggs compared with the other disinfectants. Followed by the enzymatic and hypochlorite treatments that showed a significant difference of 26.80 and 25.00, respectively, compared with the other treatments (but not with each other) (Table 2).

**Table 2:** Number of hatched *Toxocara canis* eggs, due to the effect of disinfectants at different times

***Treatments used***								
***Times (min)***	***Enzymatic***	***Ammonium***	***Hypochlorite***	***Benzalkonium***	***Iodine***	***Oxidation***	***Water***	***EE***
10	26.80^ab^	50.50^a^	25.00^ab^	10.40^b^	0.20^b^	1.60^b^	0.50^b^	6.02
20	98.80^a^	55.10^b^	17.30^c^	11.30^c^	12.70^c^	3.10^c^	1.30^c^	25.9
30	334.10^a^	65.40^b^	26.20^b^	19.00^b^	20.40^b^	5.10^b^	1.50^b^	18.9
40	381.70^a^	70.40^b^	42.70^b^	40.80^b^	19.70^b^	32.30^b^	2.50^b^	190.1
1440	546.9^a^	752.60^a^	128.0^a^	68.2^a^	83.1^a^	45.2^a^	10.8^a^	---

abc Mean with different literals present significant difference; min = minutes; EE = standard error

After 20 minutes, the enzymatic treatment showed a significant difference of 98.80 broken eggs versus ammonium and the other treatments. The ammonium treatment had fewer broken eggs than the enzymatic one, but still presented a significant difference of 55.10 broken eggs compared with the other disinfectants. In the 30 and 40-minute times, only the enzymatic treatment showed a significant difference of 334.10 and 381.70 hatched eggs, respectively, compared with all the other disinfectants.

Table 3 shows the percentages of eggs broken by times. We found that at 10 minutes the ammonium showed significant difference compared with the other treatments, obtaining the highest percentage of broken eggs (5.94%); the enzymatic treatment had a lower percentage of eggs than ammonium and had no difference compared with hypochlorite. After 20 minutes, the enzymatic treatment was higher in percentage of broken eggs (10.68%), presenting a significant difference compared with ammonium and the other treatments. In the remaining times, only the enzymatic treatment showed significant difference at 40 minutes with 44.90% broken eggs compared with all the treatments, obtaining a higher percentage of broken eggs.

**Table 3:** Percentage of hatched eggs, by the effect of disinfectants at different times

***Treatments used***									
***Times (min)***	***Enzymatic***	***Ammonium***	***Hypochlorite***	***Benzalkonium***	***Iodine***	***Oxidation***	***Water***	***EE***	***CV%***
10	3.15^ab^	5.94^a^	2.94^ab^	1.22^b^	0.24^b^	0.18^b^	0.05^b^	0.80	131.2
20	10.68^a^	6.48^b^	2.03^c^	1.32^c^	1.49^c^	0.36^c^	0.15^c^	0.70	69.6
30	39.30^a^	7.69^b^	3.08^b^	2.23^b^	2.40^b^	0.60^b^	0.17^b^	3.05	121.7
40	44.90^a^	8.28^b^	5.02^b^	4.79^b^	2.31^b^	3.79^b^	0.29^b^	2.23	71.2
1440	64.34^a^	88.54^a^	15.06^a^	8.02^a^	9.78^a^	5.32^a^	1.27^a^	22.36	257.4

abc Mean with different literals present significant difference; min = minutes; EE = standard error; CV = coefficient of variation

## Discussion

There is a variety of disinfectants that are used daily, whose effect on pathogens in this case in nematode eggs is unknown, when evaluating the effect for determined periods of time, at 10 minutes the ammonium had a significant difference of 50.50 broken eggs compared with the other disinfectants when presenting a greater number of broken eggs. Followed by the enzymatic and hypochlorite treatments, which showed a significant difference of 26.80 and 25.00 broken eggs, respectively, compared with the other treatments (but not with each other).

At 20 min, the enzymatic treatment showed a significant difference of 98.80 eggs broken compared with ammonium and the other treatments. The ammonium treatment had fewer broken eggs than the enzymatic one, but still presented a significant difference of 55.10 broken eggs compared with the other disinfectants. The results obtained differ from another (2) that treated *T. canis* eggs and observed the motility of the larvae under 40x magnification with a microscope. The eggs treated in 2.5%, 5%, 7.5%, and 10% iodine solutions were completely non-mobile after 20, 40, and 60 minutes of treatment, while the eggs treated with the other disinfectants (i.e., glutaraldehyde, benzalkonium chloride, sodium hypochlorite, and phenol) were still mobile after 24 hours. These were higher values of time compared with those obtained in the present study.

Another study (15) about the action of various disinfectants on the structure and viability of *Toxocara* eggs showed that all eggs were degenerated in a few days with the use of 70% ethanol. In a study (16) for accelerating the maturation of *T. cati* was shown that the use of sodium hypochlorite eliminated the outer layer of the eggs. However, the eggs harbor infective larvae for a maximum of 3 weeks. According to the comparison with the control group, there was no effect on the embryonation of *T. canis* with benzalkonium chloride and formaldehyde-based disinfectants. The findings of the present study indicate, percentages of hatched eggs were obtained at different times. For example, at 10 minutes, ammonium had the highest number of hatched eggs, followed by the enzymatic and hypochlorite treatments; at 20 minutes, the enzymatic treatment was higher in percentage of hatched eggs (presenting a significant difference of 10.68 hatched eggs compared with ammonium and the other treatments); in the remaining times, only the enzymatic treatment showed a significant difference of 64.34 hatched eggs compared with all treatments (obtaining a higher percentage of broken eggs). The use of formaldehyde leads to a delay in the development of the embryo (compared to sterile water). Alkyl-dimethyl-benzyl ammonium chloride, sodium hypochlorite, sodium hydroxide, sodium chloride, and 40% formic aldehyde solution are solutions used as disinfectants, germicides, and bactericides. These substances do not offer any type of food or physiological support to the eggs (8). An important fact is that researchers who study on *T. canis* should be aware that common chlorine treatment cannot inactivate its eggs and should take extra care when investigate on these egg samples.

Using commercial bleach containing 8% sodium hypochlorite is useful, since it is considered an effective disinfectant (17). But it is not commercially available for domestic use, so not all people can have access to this disinfectant.

## Conclusion

The commercial household disinfectants available like chlorine do not inactivate eggs. This study showed that the enzymatic disinfectant is the most effective against the eggs of *T. canis* to present a greater number of hatched eggs in a given time between 20 and 40 minutes, where it has its greatest ovicidal effect. However, additional studies are needed to develop other disinfecting agents with faster action to inactivate *T. canis* eggs and prevent them from developing infective larvae.
